# Effects of lipid-lowering drugs on epilepsy and its subtypes: A drug-target Mendelian randomization study

**DOI:** 10.1097/MD.0000000000048021

**Published:** 2026-03-20

**Authors:** Yan Wang, Shuo Huang

**Affiliations:** a Fengqiu County Traditional Chinese Medicine Hospital, Fengqiu, China; b Taian City Traditional Chinese Medicine Hospital, Taian, China.

**Keywords:** epilepsy, HMGCR, Mendelian randomization, NPC1L1, PCSK9

## Abstract

Statins, commonly used lipid-lowering drugs to reduce cardiovascular risk, have also been suggested to have protective effects against epilepsy. However, whether this association is causal remains unclear. We conducted a drug-target Mendelian randomization study to examine the effects of genetically predicted inhibition of 3 established lipid-lowering targets (3-hydroxy-3-methylglutaryl coenzyme A reductase [statins], Niemann-Pick C1-Like 1 [ezetimibe], and proprotein convertase subtilisin/kexin type 9 [PCSK9 inhibitors]) on epilepsy and its subtypes. The inverse-variance weighted approach served as the primary analysis, supplemented with multiple sensitivity tests to ensure robustness. Genetically proxied 3-hydroxy-3-methylglutaryl coenzyme A reductase inhibition was associated with decreased risks of epilepsy (odds ratio [OR] = 0.87; 95% confidence interval [CI]: 0.82–0.92; *P* = 1.4 × 10^‐6^) and focal epilepsy (OR = 0.83; 95% CI: 0.76–0.92; *P* = 1.5 × 10^‐4^). Inhibition of Niemann-Pick C1-Like 1 corresponded to a lower risk of generalized epilepsy with tonic−clonic seizures (OR = 0.98; 95% CI: 0.97–0.98; *P* = 1.2 × 10^‐34^) but increased risks of focal epilepsy (lesion negative, OR = 1.08; 95% CI: 1.06–1.09; *P* = 5.9 × 10^‐31^), childhood absence epilepsy (OR = 1.06; 95% CI: 1.05–1.07; *P* = 1.8 × 10^‐21^), and juvenile absence epilepsy (OR = 1.03; 95% CI: 1.01–1.05; *P* = 9.7 × 10^‐3^). PCSK9 inhibition was linked to reduced risks of generalized epilepsy with tonic−clonic seizures (OR = 0.99; 95% CI: 0.98–1.00; *P* = 1.4 × 10^‐2^), juvenile absence epilepsy (OR = 0.96; 95% CI: 0.93–1.00; *P* = 2.7 × 10^‐2^), and juvenile myoclonic epilepsy (OR = 0.96; 95% CI: 0.93–1.00; *P* = 2.9 × 10^‐2^). The effects of lipid-lowering drug targets on epilepsy risk vary by target and exhibit pleiotropy. Statins and PCSK9 inhibitors appear protective against epilepsy and several subtypes, whereas ezetimibe may increase susceptibility to certain subtypes. These results underscore the importance of considering target-specific effects when choosing lipid-lowering therapies for patients with or at risk of epilepsy.

## 1. Introduction

Epilepsy is a prevalent neurologic disorder, affecting an estimated 45.9 million individuals worldwide in 2016, and continues to contribute substantially to disability and mortality.^[1]^ Stroke is a major cause of acute symptomatic seizures and epilepsy in individuals over 65 years old.^[2]^ With the increasing incidence of stroke in aging populations, stroke seizures pose a growing clinical challenge.^[3]^ As populations continue to age post-globally, the overall prevalence of epilepsy is projected to rise further.^[4]^ Although a variety of antiepileptic drugs are available and treatment is generally tailored based on etiology, age, and seizure type, approximately 30% of patients remain resistant to standard therapies and experience recurrent seizures.^[5]^

Statins rank among the most widely prescribed medications for cardiovascular disease, used in both primary and secondary prevention of atherosclerosis.^[6]^ Beyond their cholesterol-loweringproperties, statins exhibit pleiotropic neuroprotective effects, demonstrating benefits in conditions such as multiple sclerosis and spinal cord injury.^[7,8]^ Recent evidence suggests that statins can protect cortical neurons from excitotoxicity and influence epileptogenesis, highlighting their potential role in epilepsy prevention.^[9]^ Elevated serum free cholesterol has also been linked to poorer outcomes in patients with status epilepticus, with higher levels associated with worse discharge prognoses.^[3]^ Together, these observations imply that cholesterol-lowering therapies may help attenuate excitotoxic injury and related neurological consequences. Epidemiological studies provide supporting evidence: in a population-based nested case–control study from Quebec involving 217 hospital-diagnosed epilepsy cases and 2170 matched controls, current statin users had a significantly lower risk of epilepsy (adjusted rate ratio = 0.65, 95% confidence interval [CI]: 0.46–0.92), while past users showed a trend toward reduced risk (adjusted rate ratio = 0.72, 95% CI: 0.39–1.30). Higher cumulative statin doses were associated with greater risk reduction, whereas no protective effect was observed for non-statin lipid-lowering drugs, beta-blockers, or angiotensin-converting enzyme inhibitors.^[10]^ Nevertheless, the observational design limits causal interpretation.

Mendelian randomization (MR) is a genetic epidemiology method that leverages germline variants as instrumental variables to assess causal relationships between exposures and outcomes. By exploiting the random allocation of alleles at conception, MR minimizes confounding and reverse causation, which are common limitations of observational studies. This approach has become a robust tool for causal inference in epidemiology.^[11–13]^ In the present study, we employed a drug-target MR framework to evaluate the causal effects of genetically predicted inhibition of 3 validated lipid-lowering targets (3-hydroxy-3-methylglutarylcoenzyme A reductase [HMGCR], proprotein convertase subtilisin/kexin type 9 [PCSK9], and Niemann-Pick C1-like 1 [NPC1L1]) on epilepsy and its subtypes. Our aim was to identify target-specific benefits and potential trade-offs of lipid-loweringtherapies, providing evidence to inform precision medicine strategies for individuals with or at risk of epilepsy.

## 2. Methods

### 2.1. Study design

**Figure 1.**
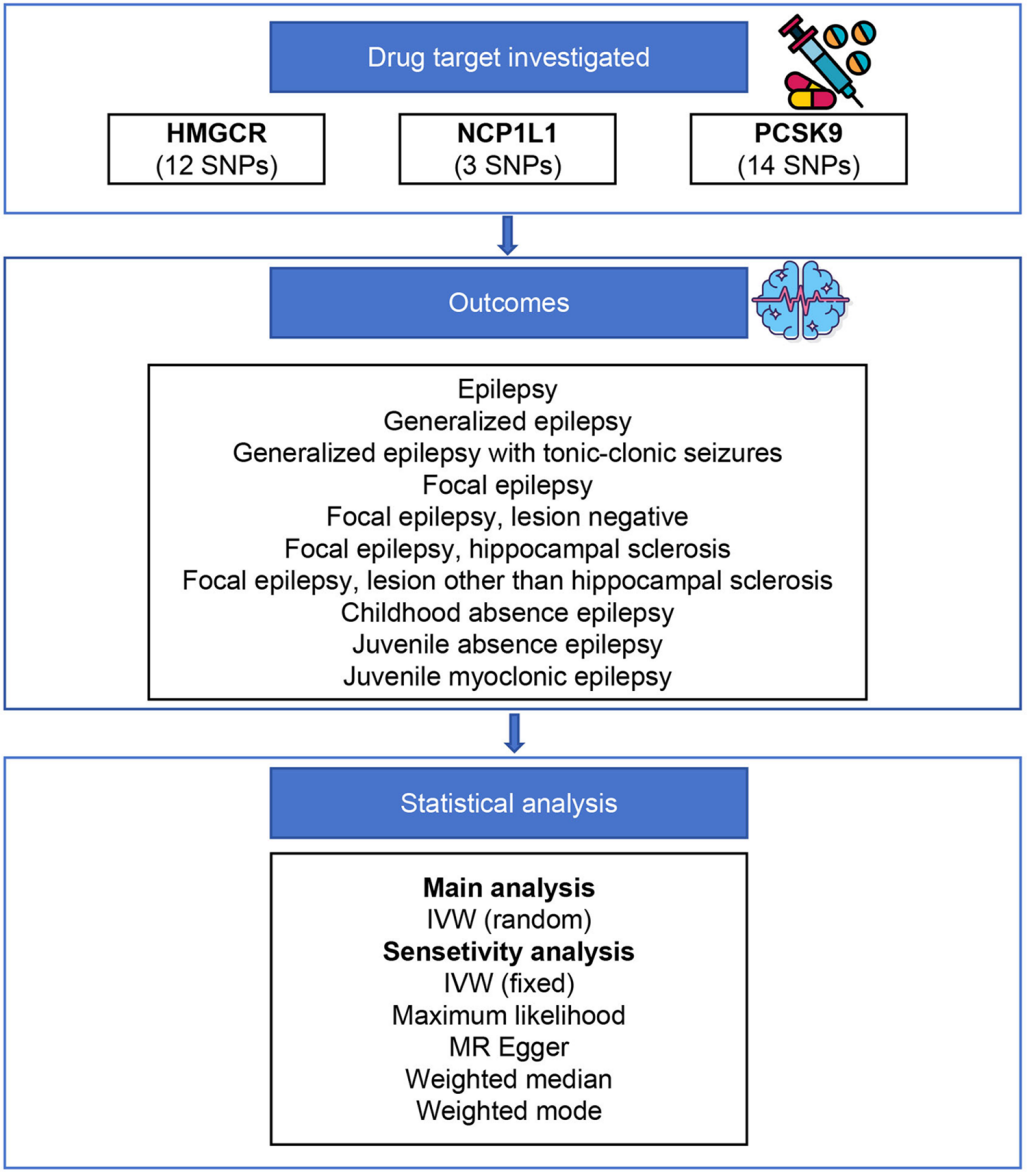
Overview of the study design. HMGCR = 3-hydroxy-3-methylglutaryl coenzyme A reductase; IVW = inverse-variance weighted; NPC1L1 = Niemann-Pick C1-Like 1; PCSK9 = proprotein convertase subtilisin/kexin type 9; SNP = single nucleotide polymorphism.

This study adhered to the STROBE-MR reporting guidelines^[14]^ and met the 3 core assumptions necessary for valid MR inference: genetic variants are strongly associated with the exposure at genome-wide significance; the variants are not linked to potential confounders of the exposure–outcome relationship; and the variants affect the outcome solely through the exposure of interest. As shown in Figure 1, we implemented a drug-target MR approach to evaluate the causal effects of genetically predicted inhibition of lipid-lowering targets (HMGCR, PCSK9, and NPC1L1) on the risk of epilepsy and its subtypes. Summary-level data for these outcomes were obtained from the International League Against Epilepsy Consortium on Complex Epilepsies.^[15]^ All epilepsy phenotypes in the genome-wide association study were assigned by epilepsy specialists based on standardized clinical evaluations and classified following the 2010 International League Against Epilepsy criteria. Phenotypic assignments relied on electroencephalogram, magnetic resonance imaging, and clinical history data. The dataset comprises 3 primary epilepsy phenotypes: all epilepsy (n = 44,889), focal epilepsy (n = 39,348), and generalized epilepsy (n = 33,446), and 7 subphenotypes; generalized epilepsy with tonic–clonic seizures (n = 29,905), focal epilepsy (lesion negative, n = 32,393), focal epilepsy (hippocampal sclerosis, n = 30,480), focal epilepsy (lesion other than hippocampal sclerosis, n = 32,747), childhood absence epilepsy (n = 30,470), juvenile absence epilepsy (n = 30,092), and juvenile myoclonic epilepsy (n = 30,858). Employing these data allowed us to reduce the confounding effects of population stratification, ensuring results relevant to European populations. Detailed information for each dataset is provided in Table S1, Supplemental Digital Content, https://links.lww.com/MD/R566.

### 2.2. Genetic instruments of LDL-lowering drug targets

Summary statistics for low-density lipoprotein cholesterol (LDL-C) were sourced from a genome-wide association study meta-analysis by the Global Lipid Genetics Consortium, which included 173,082 individuals of European ancestry.^[16]^ Single-nucleotide polymorphisms (SNPs) were selected as genetic instruments according to the following criteria: genome-wide significant association with LDL-C (*P* < 5 × 10^‐8^) and located within ± 500 kb of the gene encoding the respective drug target; low linkage disequilibrium between SNPs (*r*^2^ < 0.30); exclusion of palindromic SNPs with ambiguous strand orientation; and effect alleles associated with lower LDL-C to model pharmacological inhibition, ensuring all SNPs had negative β coefficients. Ultimately, 12 SNPs served as proxies for HMGCR inhibition, 14 for PCSK9 inhibition, and 3 for NPC1L1 inhibition (Table S2, Supplemental Digital Content, https://links.lww.com/MD/R566).

### 2.3. Statistical analysis

The primary MR analysis employed the inverse-variance weighted (IVW) method under a multiplicative random-effects model. To test the robustness of the results, we performed additional sensitivity analyses, including IVW with fixed effects, maximum likelihood, MR-Egger regression, weighted median, and weighted mode approaches. Heterogeneity among instruments was assessed using Cochran *Q* statistic, and a nonzero MR-Egger intercept was interpreted as evidence of directional pleiotropy.^[17]^ Effect estimates were presented as odds ratios (OR) with 95% CIs, and statistical significance was defined as a two-sided *P* < .05. All analyses were conducted in R (version 4.4.1; R Foundation for Statistical Computing, Vienna, Austria) using the TwoSampleMR package (version 0.6.17; MRC Integrative Epidemiology Unit, University of Bristol, Bristol, UK).

## 3. Results

### 3.1. Effects of HMGCR inhibition on epilepsy and its subtypes

**Figure 2.**
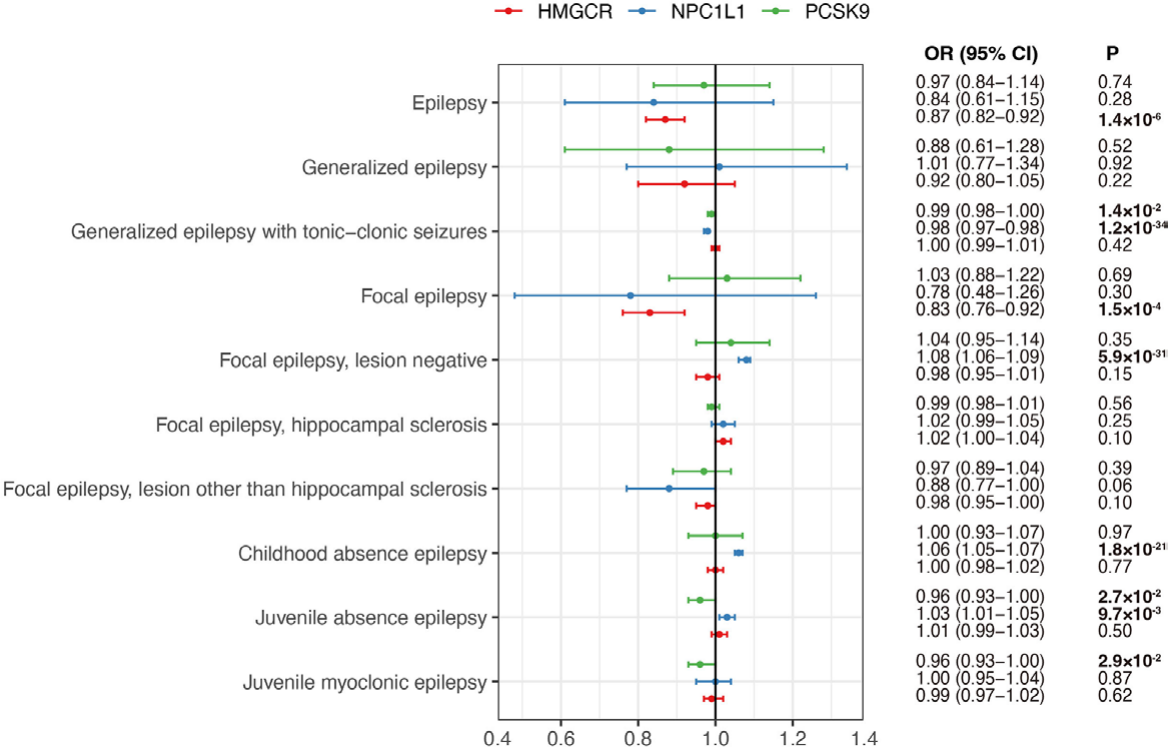
Forest plot showing the effects of genetically proxied inhibition of HMGCR, NPC1L1, and PCSK9 on epilepsy and its subtypes. CI = confidence interval; HMGCR = 3-hydroxy-3-methylglutaryl coenzyme A reductase; NPC1L1 = Niemann-Pick C1-Like 1; OR = odd ratio; PCSK9 = proprotein convertase subtilisin/kexin type 9; SNP = single nucleotide polymorphism.

In the primary IVW MR analysis, genetically proxied inhibition of HMGCR, equivalent to a 1 mmol/L (38.7 mg/dL) reduction in LDL-C, was significantly associated with lower risks of epilepsy (OR = 0.87; 95% CI: 0.82–0.92; *P* = 1.4 × 10^‐6^) and focal epilepsy (OR = 0.83; 95% CI: 0.76–0.92; *P* = 1.5 × 10^‐4^; Fig. 2). These associations remained significant using the fixed-effects IVW method (epilepsy: OR = 0.87, 95% CI: 0.78–0.96, *P* = .01; focal epilepsy: OR = 0.83, 95% CI: 0.74–0.94, *P* = 4.1 × 10^‐3^) and the maximum likelihood method (epilepsy: OR = 0.87, 95% CI: 0.78–0.97, *P* = .01; focal epilepsy: OR = 0.83, 95% CI: 0.73–0.94, *P* = 4.1 × 100^‐3^; Table 1). No significant associations were observed for other epilepsy subtypes. Cochran *Q* test indicated no heterogeneity, and MR-Egger regression suggested an absence of directional pleiotropy.

**Table 1:** Sensitivity analysis.

Outcome	IVW (fixed)		Maximum likelihood		MR Egger		Weighted median		Weighted mode		Heterogeneity	Pleiotropy
	OR (95% CI)	*P*	OR (95% CI)	*P*	OR (95% CI)	*P*	OR (95% CI)	*P*	OR (95% CI)	*P*	*P*	*P*
*HMGCR*												
Epilepsy	0.87 (0.78–0.96)	.01	0.87 (0.78–0.97)	.01	0.90 (0.64–1.25)	.54	0.89 (0.77–1.02)	.10	0.89 (0.76–1.06)	.22	.99	.83
Generalized epilepsy	0.92 (0.76–1.10)	.36	0.92 (0.76–1.10)	.37	1.11 (0.63–1.96)	.72	0.99 (0.78–1.26)	.95	0.97 (0.74–1.28)	.85	.87	.50
Generalized epilepsy with tonic-clonic seizures	1.00 (0.98–1.01)	.55	1.00 (0.98–1.01)	.55	0.97 (0.93–1.01)	.14	1.00 (0.98–1.01)	.69	1.00 (0.98–1.02)	.68	.88	.16
Focal epilepsy	0.83 (0.74-0.94)	4.1 × 10^‐3^	0.83 (0.73–0.94)	4.1 × 10^‐3^	0.82 (0.56–1.21)	.35	0.86 (0.73–1.02)	.08	0.86 (0.70–1.05)	.17	.85	.95
Focal epilepsy, lesion negative	0.98 (0.94–1.02)	.24	0.98 (0.94–1.02)	.24	0.99 (0.88–1.11)	.81	0.99 (0.94–1.04)	.76	1.00 (0.94–1.07)	.97	.75	.89
Focal epilepsy, hippocampal sclerosis	1.02 (1.00–1.04)	.08	1.02 (1.00–1.04)	.08	1.08 (1.01–1.15)	.02	1.02 (0.99–1.05)	.12	1.02 (0.99–1.06)	.18	.33	.12
Focal epilepsy, lesion other than hippocampal sclerosis	0.98 (0.94–1.02)	.24	0.98 (0.94–1.02)	.25	0.89 (0.79–1.00)	.08	0.96 (0.91–1.01)	.12	0.96 (0.90–1.02)	.23	.90	.13
Childhood absence epilepsy	1.00 (0.97–1.02)	.80	1.00 (0.97–1.02)	.80	0.97 (0.90–1.04)	.37	1.00 (0.97–1.03)	.96	1.01 (0.97–1.05)	.81	.67	.39
Juvenile absence epilepsy	1.01 (0.99–1.02)	.45	1.01 (0.99–1.02)	.45	1.02 (0.96–1.09)	.50	1.02 (0.99–1.04)	.14	1.02 (1.00–1.04)	.15	.22	.62
Juvenile myoclonic epilepsy	0.99 (0.97–1.02)	.62	0.99 (0.97–1.02)	.62	1.08 (0.99–1.17)	.12	1.00 (0.96–1.04)	.88	1.01 (0.96–1.07)	.62	.47	.08
*NPC1L1*												
Epilepsy	0.84 (0.60–1.18)	.31	0.84 (0.59–1.18)	.31	–	–	–	–	–	–	.35	–
Generalized epilepsy	1.01 (0.57–1.80)	.96	1.01 (0.57–1.80)	.96	–	–	–	–	–	–	.63	–
Generalized epilepsy with tonic-clonic seizures	0.98 (0.94–1.02)	.26	0.98 (0.94–1.02)	.26	–	–	–	–	–	–	.93	–
Focal epilepsy	0.78 (0.52–1.15)	.21	0.77 (0.52–1.16)	.21	–	–	–	–	–	–	.23	–
Focal epilepsy, lesion negative	1.08 (0.96–1.21)	.21	1.08 (0.96–1.21)	.21	–	–	–	–	–	–	.91	–
Focal epilepsy, hippocampal sclerosis	1.02 (0.95–1.09)	.58	1.02 (0.95–1.09)	.58	–	–	–	–	–	–	.63	–
Focal epilepsy, lesion other than hippocampal sclerosis	0.88 (0.78–0.99)	.03	0.88 (0.78–0.99)	.03	–	–	–	–	–	–	.26	–
Childhood absence epilepsy	1.06 (0.99–1.14)	.11	1.06 (0.99–1.14)	.11	–	–	–	–	–	–	.87	–
Juvenile absence epilepsy	1.03 (0.97–1.08)	.36	1.03 (0.97–1.08)	.36	–	–	–	–	–	–	.72	–
Juvenile myoclonic epilepsy	1.00 (0.92–1.08)	.93	1.00 (0.92–1.08)	.93	–	–	–	–	–	–	.58	–
*PCSK9*												
Epilepsy	0.97 (0.83–1.14)	.74	0.97 (0.83–1.14)	.74	1.34 (0.65–2.74)	.48	0.91 (0.75–1.10)	.33	0.90 (0.73–1.12)	.41	.43	.44
Generalized epilepsy	0.88 (0.68–1.15)	.37	0.88 (0.68–1.15)	.36	0.50 (0.08–3.18)	.52	0.75 (0.54–1.06)	.10	0.69 (0.45–1.07)	.18	.10	.58
Generalized epilepsy with tonic-clonic seizures	0.99 (0.97–1.01)	.30	0.99 (0.97–1.01)	.30	0.99 (0.91–1.08)	.85	0.99 (0.97–1.01)	.29	0.98 (0.96–1.01)	.33	.95	.99
Focal epilepsy	1.03 (0.86–1.24)	.72	1.03 (0.86–1.24)	.72	1.63 (0.71–3.73)	.33	0.99 (0.79–1.23)	.91	0.98 (0.73–1.33)	.92	.51	.35
Focal epilepsy, lesion negative	1.04 (0.99–1.10)	.12	1.05 (0.99–1.10)	.12	0.87 (0.57–1.35)	.59	1.03 (0.95–1.12)	.42	1.16 (1.04–1.29)	6.9 × 10^‐3^	.03	.47
Focal epilepsy, hippocampal sclerosis	0.99 (0.96–1.03)	.73	0.99 (0.96–1.03)	.73	1.00 (0.86–1.15)	.96	0.99 (0.96–1.03)	.77	1.00 (0.95–1.05)	.89	.84	.99
Focal epilepsy, lesion other than hippocampal sclerosis	0.97 (0.91–1.02)	.22	0.97 (0.91–1.02)	.22	1.17 (0.82–1.67)	.44	0.92 (0.85–0.99)	.03	0.91 (0.81–1.03)	.22	.09	.35
Childhood absence epilepsy	1.00 (0.97–1.03)	.94	1.00 (0.97–1.03)	.94	0.81 (0.60–1.10)	.27	0.97 (0.93–1.01)	.18	0.94 (0.90–0.99)	.01	5.8 × 10^‐4^	.26
Juvenile absence epilepsy	0.96 (0.94–0.99)	4.1 × 10^‐3^	0.96 (0.94–0.99)	4.2 × 10^‐3^	0.90 (0.77–1.04)	.26	0.97 (0.94–1.01)	.11	0.98 (0.93–1.03)	.43	.15	.42
Juvenile myoclonic epilepsy	0.96 (0.93–1.00)	.06	0.96 (0.93–1.00)	.06	1.02 (0.85–1.22)	.87	0.97 (0.93–1.02)	.27	0.98 (0.92–1.03)	.45	.56	.59

CI = confidence interval, HMGCR = 3-hydroxy-3-methylglutaryl coenzyme A reductase, IVW = inverse-variance weighted, MR = Mendelian randomization, NPC1L1 = Niemann-Pick C1-Like 1, OR = odd ratio, PCSK9 = proprotein convertase subtilisin/kexin type 9.

### 3.2. Effects of NPC1L1 inhibition on epilepsy and its subtypes

In the IVW random-effects analysis, genetically proxied inhibition of NPC1L1 was associated with a lower risk of generalized epilepsy with tonic–clonic seizures (OR = 0.98; 95% CI: 0.97–0.98; *P* = 1.2 × 10^-34^) but increased risks of focal epilepsy (lesion negative, OR = 1.08; 95% CI: 1.06–1.09; *P* = 5.9 × 10^‐31^), childhood absence epilepsy (OR = 1.06; 95% CI: 1.05–1.07; *P* = 1.8 × 10^‐21^), and juvenile absence epilepsy (OR = 1.03; 95% CI: 1.01–1.05; *P* = 9.7 × 10^‐3^; Fig. 2). Cochran *Q* test did not indicate significant heterogeneity (Table 1).

### 3.3. Effects of PCSK9 inhibition on epilepsy and its subtypes

In the primary IVW MR analysis, genetically proxied inhibition of PCSK9 was significantly associated with reduced risks of generalized epilepsy with tonic−clonic seizures (OR = 0.99; 95% CI: 0.98–1.00; *P* = 1.4 × 10^‐2^), juvenile absence epilepsy (OR = 0.96; 95% CI: 0.93–1.00; *P* = 2.7 × 10^‐2^), and juvenile myoclonic epilepsy (OR = 0.96; 95% CI: 0.93–1.00; *P* = 2.9 × 10^‐2^). The association with juvenile absence epilepsy was confirmed by both the fixed-effects IVW method (OR = 0.96; 95% CI: 0.94–0.99; *P* = 4.1 × 10^‐3^) and the maximum likelihood method ((OR = 0.96; 95% CI: 0.94–0.99; *P* = 4.2 × 10^‐3^). Cochran *Q* test revealed significant heterogeneity for PCSK9 inhibition with focal epilepsy (lesion negative) and childhood absence epilepsy, whereas MR-Egger regression indicated no evidence of directional pleiotropy.

### 3.4. Overall summary

**Figure 3.**
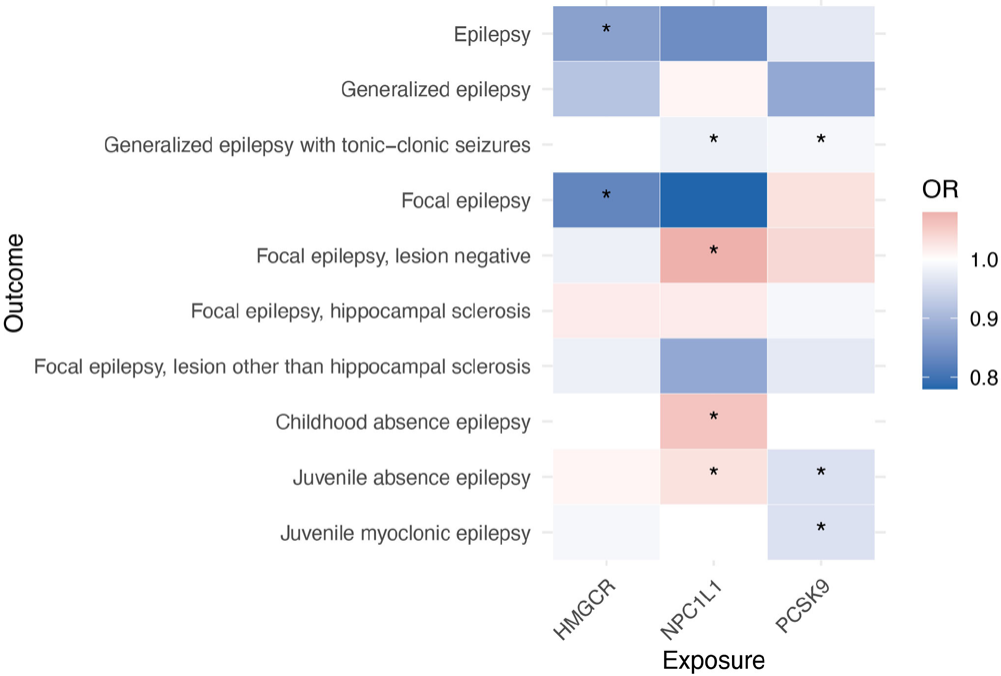
Heatmap showing the effects of genetically proxied inhibition of HMGCR, NPC1L1, and PCSK9 on epilepsy and its subtypes. HMGCR = 3-hydroxy-3-methylglutaryl coenzyme A reductase; NPC1L1 = Niemann-Pick C1-Like 1; PAD = peripheral artery disease; PCSK9 = proprotein convertase subtilisin/kexin type 9. * indicated *P* < .05.

As summarized in Figure 3 (heatmap), HMGCR inhibition was protective against overall and focal epilepsy, NPC1L1 inhibition showed both protective and harmful effects depending on the subtype, and PCSK9 inhibition conferred protection against several generalized epilepsy subtypes.

## 4. Discussion

In this two-sample MR study, we systematically evaluated the associations between genetically predicted inhibition of HMGCR, NPC1L1, and PCSK9 and the risk of epilepsy and its subtypes. Our results indicated that HMGCR inhibition reduced the risk of overall epilepsy and focal epilepsy, whereas PCSK9 inhibition was protective against generalized epilepsy with tonic–clonic seizures, juvenile absence epilepsy, and juvenile myoclonic epilepsy. Conversely, NPC1L1 inhibition lowered the risk of generalized epilepsy with tonic–clonic seizures but increased the susceptibility to several absence and focal epilepsy subtypes.

Statins are well-established cholesterol-lowering agents for hypercholesterolemia and atherosclerotic cardiovascular disease.^[6]^ Beyond lipid lowering, they exert pleiotropic effects including immunomodulation, anti-inflammation, and neuroprotection, which may be relevant in neurological disorders such as epilepsy.^[18]^ Experimental studies support these effects: atorvastatin mitigated pentylenetetrazol-induced seizures in mice and exhibited long-term antiepileptogenic activity in WAG/Rij rats, a genetic model of absence epilepsy.^[9,19]^ In models of temporal lobe epilepsy, atorvastatin and lovastatin reduced seizure frequency and excitotoxicity,^[20]^ while atorvastatin also prevented hippocampal neuronal death induced by quinolinic acid-related seizures.^[21]^ Atorvastatin has additionally been shown to enhance the efficacy of conventional antiepileptic drugs.^[18]^ Clinical evidence aligns with these experimental findings. In a Taiwanese cohort of 7435 patients with intracranial hemorrhage followed for a median of 17.6 months, statin use was associated with a reduced risk of poststroke epilepsy (adjusted hazard ratio = 0.62; 95% CI: 0.42–0.90; *P* = .01), with stronger protective effects observed in moderate-to-high-intensity statin users (adjusted hazard ratio = 0.37; 95% CI: 0.18–0.75; *P* = .01).^[22]^ Similarly, retrospective analyses in elderly veterans and other populations demonstrated that statin therapy lowered the risk of new-onset epilepsy.^[23–25]^ Meta-analytic data further confirm these protective associations (relative risk = 0.36; 95% CI: 0.25–0.53; *P* = .001).^[26]^ Collectively, these findings support a neuroprotective role for statins, consistent with our MR results.

However, some studies reported neutral or even adverse effects. Propensity score-matched cohorts showed no significant association between statin use and epilepsy incidence,^[27]^ while case reports noted occasional new-onset seizures linked to statin therapy.^[28]^ Potential explanations include selection bias, as clinicians may avoid prescribing statins to patients with epilepsy to simplify treatment or reduce drug interactions.^[29]^ Confounding by indication, or the “healthy user effect,” may also contribute, as patients on statins often engage more in preventive care, potentially lowering observed epilepsy risk independent of drug effects. These considerations highlight the limitations of observational studies and underscore the utility of MR for causal inference.

Several mechanisms may underlie the protective effects of statins. First, stroke-related neuroprotection: stroke is a leading cause of epilepsy in older adults, particularly focal epilepsy, and statins have documented benefits in acute ischemic stroke.^[30–33]^ Second, anti-inflammatory effects: statins reduce proinflammatory cytokines such as interleukin (IL)-1β, tumor necrosis factor-α and IL-6 and increase anti-inflammatory cytokines (IL-10), potentially mitigating epileptogenesis.^[34,35]^ Third, blood–brain barrier (BBB) stabilization: statins enhance BBB integrity and limit leukocyte infiltration, which may reduce seizure susceptibility.^[36]^ Fourth, attenuation of gliosis and apoptosis: statins inhibit reactive astrogliosis, modulate apoptotic pathways (Bax, Bcl-2, and Akt), and promote neuronal survival, reducing hippocampal neurodegeneration.^[37–39]^ Fifth, modulation of excitotoxicity: statins decrease N-methyl-D-aspartate receptor-mediated excitotoxicity, and dyslipidemia itself may contribute to seizure risk.^[40–43]^ Both lipophilic (e.g., atorvastatin, simvastatin, and lovastatin) and hydrophilic (e.g., pravastatin and rosuvastatin) statins appear neuroprotective, suggesting complementary vascular, anti-inflammatory, and neuronal mechanisms collectively limit epileptogenesis.

PCSK9 inhibitors and ezetimibe are newer lipid-lowering agents with less established neurological safety profiles. Ezetimibe inhibits intestinal cholesterol absorption via NPC1L1 blockade, and our MR analysis indicated a mixed effect: reduced risk of generalized epilepsy with tonic–clonic seizures but increased risks of focal and absence epilepsy subtypes. These findings imply heterogeneous effects on neural excitability and epileptogenesis, warranting further mechanistic studies. PCSK9 inhibition was associated with protection against specific generalized epilepsy subtypes, suggesting potential neuroprotective benefits, though preclinical data are limited. These results highlight that different lipid-lowering targets can exert distinct and sometimes opposing effects on epilepsy risk, emphasizing a target-specific approach in therapy selection.

Clinically, our findings suggest that statins and PCSK9 inhibitors are unlikely to increase seizure risk and may confer neuroprotection in patients with epilepsy. Conversely, NPC1L1 inhibition may require caution in patients at high seizure risk. Overall, the data support individualized, target-specific lipid-lowering strategies and inform risk-benefit discussions integrating both cardiovascular and neurological outcomes.

### 4.1. Limitations

Several limitations should be noted. First, we could not stratify by statin type or dosage, which may affect BBB penetration and neuroprotective efficacy.^[44]^ Second, epilepsy data were derived solely from individuals of European ancestry, limiting generalizability. Third, MR estimates reflect lifelong genetic exposure and may not fully capture short- or medium-term pharmacological effects. Despite these limitations, the drug-target MR framework reduces confounding and reverse causation, providing complementary evidence to observational studies. Future prospective studies across diverse populations with detailed treatment information are warranted.

## 5. Conclusion

In summary, genetically predicted inhibition of HMGCR reduced the risk of overall and focal epilepsy, while PCSK9 inhibition lowered the risk of several generalized epilepsy subtypes. NPC1L1 inhibition showed a mixed pattern, decreasing the risk of generalized epilepsy with tonic–clonic seizures but increasing susceptibility to focal and absence subtypes. These findings demonstrate the pleiotropic and target-specific effects of lipid-lowering therapies on epilepsy risk and highlight the importance of considering neurological outcomes in therapy selection. Future large-scale studies and clinical trials are needed to validate these results and inform precision medicine approaches integrating both cardiovascular and neurological outcomes.

## Author contributions

**Conceptualization:** Shuo Huang, Yan Wang.

**Data curation:** Shuo Huang, Yan Wang.

**Formal analysis:** Shuo Huang, Yan Wang.

**Funding acquisition:** Shuo Huang, Yan Wang.

**Investigation:** Shuo Huang, Yan Wang.

**Methodology:** Shuo Huang, Yan Wang.

**Project administration:** Shuo Huang, Yan Wang.

**Resources:** Shuo Huang, Yan Wang.

**Software:** Shuo Huang, Yan Wang.

**Supervision:** Shuo Huang, Yan Wang.

**Validation:** Shuo Huang, Yan Wang.

**Visualization:** Shuo Huang, Yan Wang.

**Writing – original draft:** Shuo Huang, Yan Wang.

**Writing – review & editing:** Shuo Huang.

## Supplementary Material


