# Supplementary material for: Effects of lipid-lowering drugs on epilepsy and its subtypes: A drug-target Mendelian randomization study
Source: Medicine (Baltimore). 2026 Mar 20;105(12):e48021. doi: 10.1097/MD.0000000000048021 (PMC13008212; doi:10.1097/MD.0000000000048021)
Supplement: Supplementary file 1 [file medi-105-e48021-s001.docx]

**Supplementary Table 1. Baseline characteristics of included GWAS**

| **Trait** | **Year** | **GWAS ID** | **Sample size** | **ncase** | **ncontrol** | **Number of SNPs** |
| --- | --- | --- | --- | --- | --- | --- |
| LDL-C | 2013 | ieu-a-300 | 173,082 | - | - | 2,437,752 |
| Epilepsy | 2018 | ieu-b-8 | 44,889 | 15,212 | 29,677 | 4,880,492 |
| Generalized epilepsy | 2018 | ieu-b-9 | 33,446 | 3,769 | 29,677 | 4,867,068 |
| Generalized epilepsy with tonic-clonic seizures | 2018 | ieu-b-16 | 29,905 | 228 | 29,677 | 4,988,035 |
| Focal epilepsy | 2018 | ieu-b-10 | 39,348 | 9,671 | 29,677 | 4,862,782 |
| Focal epilepsy, lesion negative | 2018 | ieu-b-11 | 32,393 | 2,716 | 29,677 | 4,988,872 |
| Focal epilepsy, hippocampal sclerosis | 2018 | ieu-b-14 | 30,480 | 803 | 29,677 | 4,948,714 |
| Focal epilepsy, lesion other than hippocampal sclerosis | 2018 | ieu-b-15 | 32,747 | 3,070 | 29,677 | 4,987,264 |
| Childhood absence epilepsy | 2018 | ieu-b-13 | 30,470 | 793 | 29,677 | 4,979,765 |
| Juvenile absence epilepsy | 2018 | ieu-b-12 | 30,092 | 415 | 29,677 | 4,986,340 |
| Juvenile myoclonic epilepsy | 2018 | ieu-b-17 | 30,858 | 1,181 | 29,677 | 4,983,225 |

GWAS: genome-wide association study; LDL-C: low-density lipoprotein cholesterol; SNP: single nucleotide polymorphism

**Supplementary Table 2. Baseline characteristics of included SNP for HMGCR, NPC1L1, and PCSK9.**

| SNP | Chr | Position | EA | OA | EAF | Beta | SE | P | F |
| --- | --- | --- | --- | --- | --- | --- | --- | --- | --- |
| HMGCR |  |  |  |  |  |  |  |  |  |
| rs4382144 | 5 | 74274675 | A | G | 0.5871 | -0.0289 | 0.0037 | 3.25E-15 | 61 |
| rs3935470 | 5 | 74352180 | G | A | 0.3839 | -0.0439 | 0.0039 | 1.59E-27 | 127 |
| rs2035191 | 5 | 74413713 | C | T | 0.1860 | -0.0611 | 0.0047 | 5.70E-35 | 169 |
| rs3857388 | 5 | 74620377 | C | T | 0.1280 | -0.0421 | 0.0059 | 2.20E-11 | 51 |
| rs10515198 | 5 | 74641560 | A | G | 0.1029 | -0.0599 | 0.0061 | 5.99E-22 | 96 |
| rs12916 | 5 | 74656539 | C | T | 0.4314 | -0.0733 | 0.0038 | 7.79E-78 | 372 |
| rs72633976 | 5 | 74907260 | A | G | 0.1293 | -0.0625 | 0.0075 | 5.20E-15 | 69 |
| rs16872670 | 5 | 74929312 | A | G | 0.0567 | -0.0553 | 0.0084 | 1.80E-09 | 43 |
| rs7717355 | 5 | 74951901 | A | G | 0.1293 | -0.0453 | 0.0061 | 2.32E-13 | 55 |
| rs10056022 | 5 | 74969415 | A | G | 0.0858 | -0.0424 | 0.0066 | 1.87E-10 | 41 |
| rs2307111 | 5 | 75003678 | C | T | 0.4116 | -0.0416 | 0.0037 | 7.34E-28 | 126 |
| rs7727150 | 5 | 75132546 | G | T | 0.4789 | -0.0240 | 0.0038 | 4.97E-09 | 40 |
| NPC1L1 |  |  |  |  |  |  |  |  |  |
| rs2073547 | 7 | 44582331 | G | A | 0.1939 | -0.0485 | 0.0049 | 1.92E-21 | 98 |
| rs217386 | 7 | 44600695 | G | A | 0.5923 | -0.0363 | 0.0038 | 1.20E-19 | 91 |
| rs7791240 | 7 | 44602589 | C | T | 0.0910 | -0.0425 | 0.0065 | 1.84E-10 | 43 |
| PCSK9 |  |  |  |  |  |  |  |  |  |
| rs2479394 | 1 | 55486064 | G | A | 0.2850 | -0.0386 | 0.0041 | 1.58E-19 | 89 |
| rs11206510 | 1 | 55496039 | T | C | 0.8456 | -0.0831 | 0.0050 | 2.38E-53 | 276 |
| rs2479409 | 1 | 55504650 | G | A | 0.3325 | -0.0642 | 0.0041 | 2.51E-50 | 245 |
| rs4927193 | 1 | 55509872 | T | C | 0.8694 | -0.0352 | 0.0056 | 4.27E-11 | 40 |
| rs11206514 | 1 | 55516004 | A | C | 0.6108 | -0.0507 | 0.0041 | 9.95E-33 | 153 |
| rs572512 | 1 | 55517344 | T | C | 0.3456 | -0.0478 | 0.0047 | 5.31E-26 | 103 |
| rs2495477 | 1 | 55518467 | A | G | 0.6060 | -0.0640 | 0.0054 | 7.28E-30 | 140 |
| rs585131 | 1 | 55524116 | T | C | 0.8153 | -0.0637 | 0.0050 | 2.70E-35 | 162 |
| rs1475701 | 1 | 55638546 | C | T | 0.0356 | -0.0904 | 0.0092 | 1.46E-20 | 97 |
| rs4927207 | 1 | 55713628 | G | A | 0.8285 | -0.0692 | 0.0049 | 2.36E-39 | 199 |
| rs2647281 | 1 | 55724704 | G | A | 0.0554 | -0.0589 | 0.0095 | 2.27E-09 | 38 |
| rs6662286 | 1 | 55730327 | C | T | 0.9063 | -0.0989 | 0.0073 | 6.30E-36 | 184 |
| rs1874776 | 1 | 55743519 | C | T | 0.7823 | -0.0440 | 0.0044 | 2.76E-21 | 100 |
| rs207145 | 1 | 55808143 | T | C | 0.8905 | -0.0495 | 0.0057 | 6.19E-18 | 75 |

EA: effect allele; HMGCR: 3-hydroxy-3-methylglutaryl coenzyme A reductase; NPC1L1: Niemann-Pick C1-Like 1; OA: other allele; PCSK9: proprotein convertase subtilisin/kexin type 9; SNP: single nucleotide polymorphism
